# Editorial: Clinicopathological factors and staging in gastrointestinal cancers

**DOI:** 10.3389/fonc.2023.1193216

**Published:** 2023-04-05

**Authors:** Ravindra Deshpande

**Affiliations:** Department of Cancer Biology, Wake Forest University School of Medicine, Winston-Salem, NC, United States

**Keywords:** gastric cancer, TNM system, prognosis, therapy, treatment

Gastric cancer (GC) represents the most prevalent malignancy in the globe with second highest mortality (1). GC to occur more frequently in men than women with average age over 60 with advanced stage at diagnosis making the prognosis poor (2). The incidence of GC observed to decline in last few decades and this is mainly attributed to decreased prevalence of *Helicobacter Pylori* infection and change in lifestyle habits such as smoking, food storage and preservation (3). GC patients are treated with surgery. The choice of surgical resection affected by few cofounding factors such as histological grade of tumor, location and TNM category. Irrespective of this, relapse is observed in most of the patients (4). On treatment front, neoadjuvant chemotherapy in combination with radiation is chosen to prevent the tumor dissemination (5). In GC with locally advanced and metastatic form, patients treated with modified approaches such as targeted therapy, and immunotherapy. At the same time, more advanced approaches such as tumor vaccines, adoptive cell therapy are pondered (6). The proposed ways curated to achieve greater clinical benefit with personal and comprehensive diagnosis.

Gastric cancer diagnosis is based on invasion extent of primary tumor (T-stage), lymph node status (N-stage), and distant spread (M-stage), also known as TNM system (7). However, this system is not perfect and additional parameters such as distant spread, tumor micro environmental markers, treatment effects and circulating biomarkers viewed as attractive targets. In view of this, the Research Topic ‘Clinicopathological Factors and Staging in Gastrointestinal Cancers’ sheds light on four important aspects including, the refinement of GC staging system; prognostic prediction based on GCstaging; survival analyses of clinicopathological factors in GC and treatment choices in GC.

A total of 09 articles published on refinement of staging system (Wu et al, Ren et al., Zhang et al., Liu et al., Bao et al., Hongkun et al., Jeon et al., Yue et al.),. Among these reports, Ren et al describes the retrospective study to use of immunoscores to distinguish the outcomes in stage II and III colorectal cancer. Zhang et al. proposes to use the Lymph node metastasis (LNM) based staging system to predict prognosis in ampullary carcinoma (AC) cases where as Wu et al., note that the overall survival was comparable in N0 stage with N1 stage when less than 8 lymph nodes were present and therefore proposes to give emphasis in stage II colon cancer with less than 8 lymph nodes. Jeon et al., compares the surgical outcomes in surgical and pathological stages of colon cancer patients and infers that there is significant difference exists between surgical T4 and T3 in pathologic stage IIA.

Prognosis in gastric cancer patients with identical TNM stage and receiving the similar treatment is observed to be different (8), presenting the need of personalized biomarkers and tailored therapies to yield clinically significant results. Pathomics signature derived from hematoxylin and eosin stained slides of gastric cancer patients to assign prognostic benefit associated with adjuvant chemotherapy (9). In present topic, 17 articles have shed light on the refinement of existing system to accurately predict prognosis (Cheng et al., Bae at al., Gu et al., Huang at al., Hu at al., Tang and Chen, Chen at al., Li et al., Li et al., Marano et al., Li et al., Lu et al., Liu et al., Zheng et al., Chen et al., Zheng et al., Wang et al.),. The published studies propose to use expression pattern of uniquely expressed genes to understand the clinicopathological features and accurately predict the survival. For instance, Wang et al., observed that USP22 expression in gastric cancer tissues is associated with lymph node and distant metastasis and TNM grade. Zheng et al., Lu et al. perform data analysis from the TCGA database to identify OAS1 to be overexpressed in pancreatic cancer tissue and associated with poor survival. Authors also conform the OAS1 expression in pathological tissues and note that apoptosis, Notch signaling and p53 pathways were associated with OAS1 expression. On similar note, Li et al. examines the expression of SOX30 in 195 CRC and adjacent tissues and observe that its expression was decreased in CRC tissue and negatively associated with tumor size and lymph node metastasis. This collection also features 04 review and systematic review of meta analysis articles (Kinami et al., Jiang et al., Váncsa et al., Yue et al.),. Kinami et al. summarizes the importance of determining lymph node metastasis in diagnosis of gastric cancer. Xi Jiang et al. reviews the anti-tumor effects of berberine (BBR) in CRC. Authors note that BBR exerts antitumor effect in CRC by regulating microbiota and mucosal barrier function and attest to use this drug for chemotherapy. In addition, Zhang et al., attempts to develop prediction model using vascular endothelial growth factor receptor 2 (VEGFR2) immunohistochemistry in 206 HCC patients. Authors also uses contrast-enhanced MRI parameters in predicting VEGFR2 expression. In another interesting study, Gao et al., uses bioinformatics approaches to examine the DNA methylation, copy number variation and regulated miRNAs of Gamma-aminobutyric acid transaminase (ABAT) in HCC. Author’s infer that ABAT expression was lower in HCC and miR-135a-5p may be the upstream regulatory miRNA for ABAT. Immunotherapy is well recognized treatment for HCC and know to improve therapeutic response. Presently, one of the attractive strategy is to use tyrosine kinase inhibitors synergizing with immune checkpoint inhibitors including anti-PD-1/PD-L1 (10). Zheng et al., screens 880 drugs to identify Anlotinib downregulates PD-L1 expression and may benefit with anti PD-1 inhibitor in clinical setting for GC.

Together, the brief overview of the articles presented here provide the glimpse of updates on use of prognostic and clinicopathological factors in affecting the gastric cancer staging and eventually the treatment course.

## Author contributions

The author confirms being the sole contributor of this work and has approved it for publication.
